# Supplementary material for: Multiparity and Aging Impact Chondrogenic and Osteogenic Potential at Symphyseal Enthesis: New Insights into Interpubic Joint Remodeling
Source: Int J Mol Sci. 2023 Feb 26;24(5):4573. doi: 10.3390/ijms24054573 (PMC10003663; doi:10.3390/ijms24054573)
Supplement: Supplementary file 1 [file ijms-24-04573-s001.zip › ijms-2164252-supplementary.pdf]

Supplemental table S1 – Primers Sequences

Table S1. Sequences of primers used in the present work.

Gene	Primer sequence	Amplicon	Reference Sequence NCBI
36b4	F: 5’- GACCACCAAGACATACGGGG-3'<br>R: 5'- AGTGTGCGATGCTCTGGTAG-3'	196	NM_007927.3
Sox-9	F: 5’-TTCAGATGCAGTGAGGAGCA-3’<br>R: 5’-TATCCACGGCACACACTT-3’	140	NM_011448.4
Runx-2	F: 5’- CCCAGCCACCTTTACCTACA-3’<br>R: 5’- TATGGAGTGCTGCTGGTCTG-3’	149	NM_001146038.3
Col2a1	F: 5’- CATCGACATGTCAGCCTTTG-3’<br>R: 5’- CGGATGCTCTCAATCTGGTT-3’	158	NM_031163.3
